# Supplementary material for: Curcumin Derivative CU4c Exhibits HDAC-Inhibitory and Anticancer Activities Against Human Lung Cancer Cells In Vitro and in Mouse Xenograft Models
Source: Pharmaceuticals (Basel). 2025 Jun 26;18(7):960. doi: 10.3390/ph18070960 (PMC12297909; doi:10.3390/ph18070960)
Supplement: Supplementary file 1 [file pharmaceuticals-18-00960-s001.zip › pharmaceuticals-3703757-supplementary.pdf]

Supplementary materials

Article

Curcumin Derivative CU4c Exhibits HDAC Inhibitory and Anticancer Activities against Human Lung Cancer Cells in vitro and in Mouse Xenograft Models

Narissara Namwan, Gulsiri Senawong, Chanokbhorn Phaosiri, Pakit Kumboonma, La-or Somsakeesit, Pitchakorn Sangchang and Thanaset Senawong

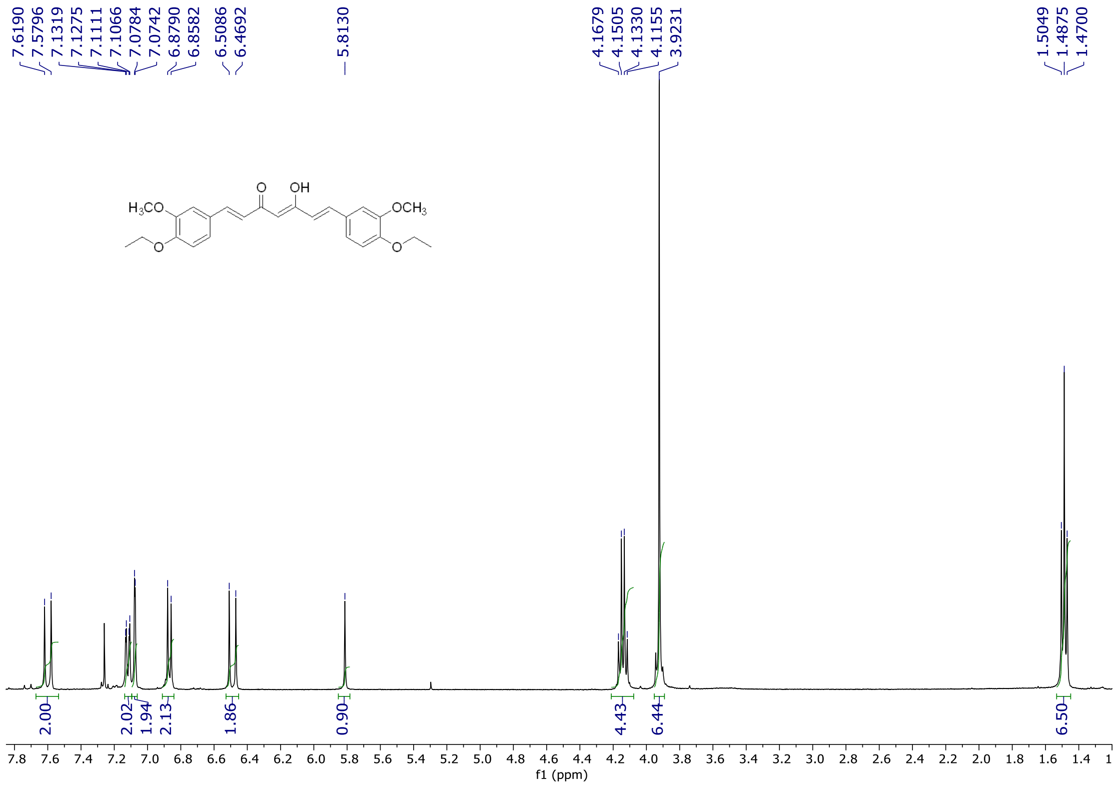

Figure S1 ^{1}H NMR spectrum of CU4c.

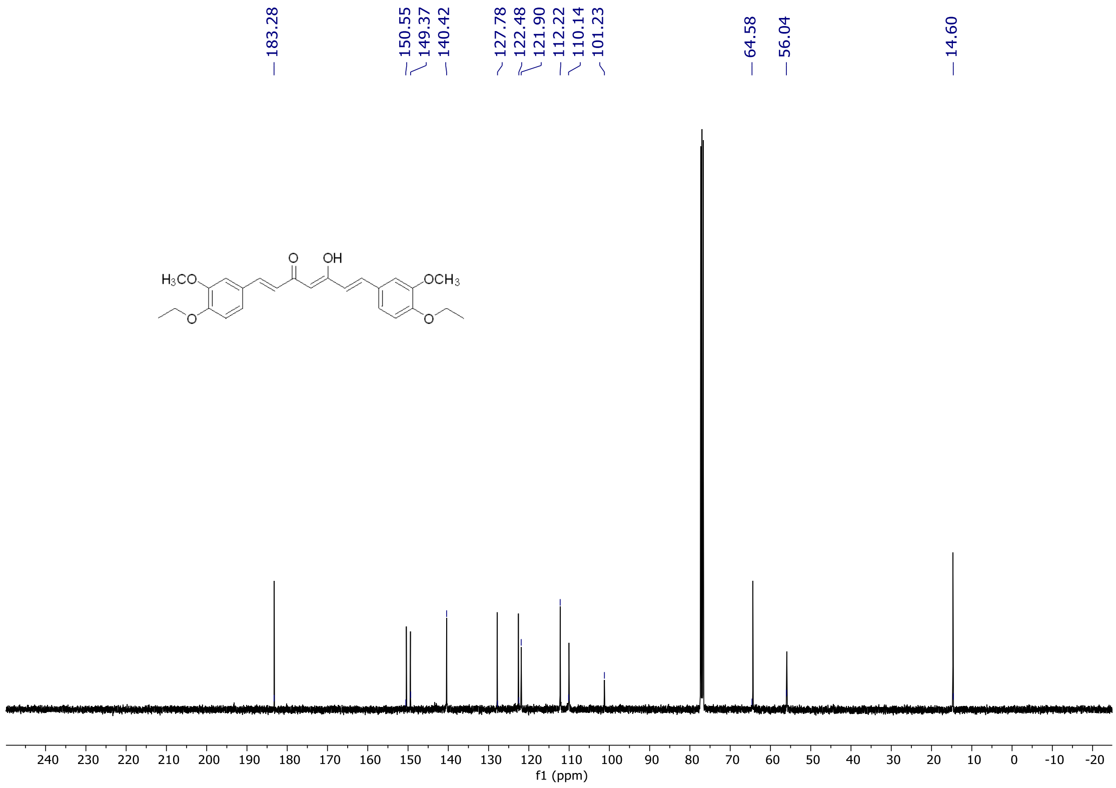

Figure S2 ^{13}C NMR spectrum of CU4c.

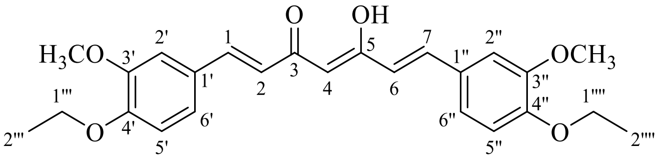

Figure S3 Chemical structure of CU4c.

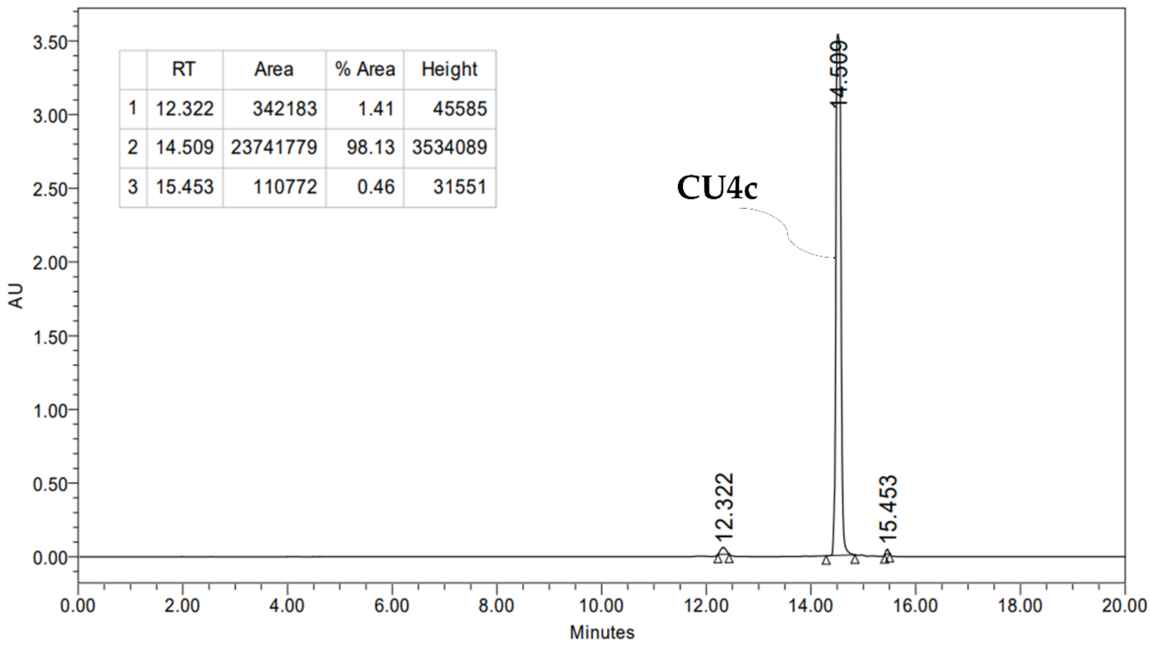

	RT	Area	% Area	Height
1	12.322	342183	1.41	45585
2	14.509	23741779	98.13	3534089
3	15.453	110772	0.46	31551

Figure S4 HPLC profile of CU4c at 100 ppm

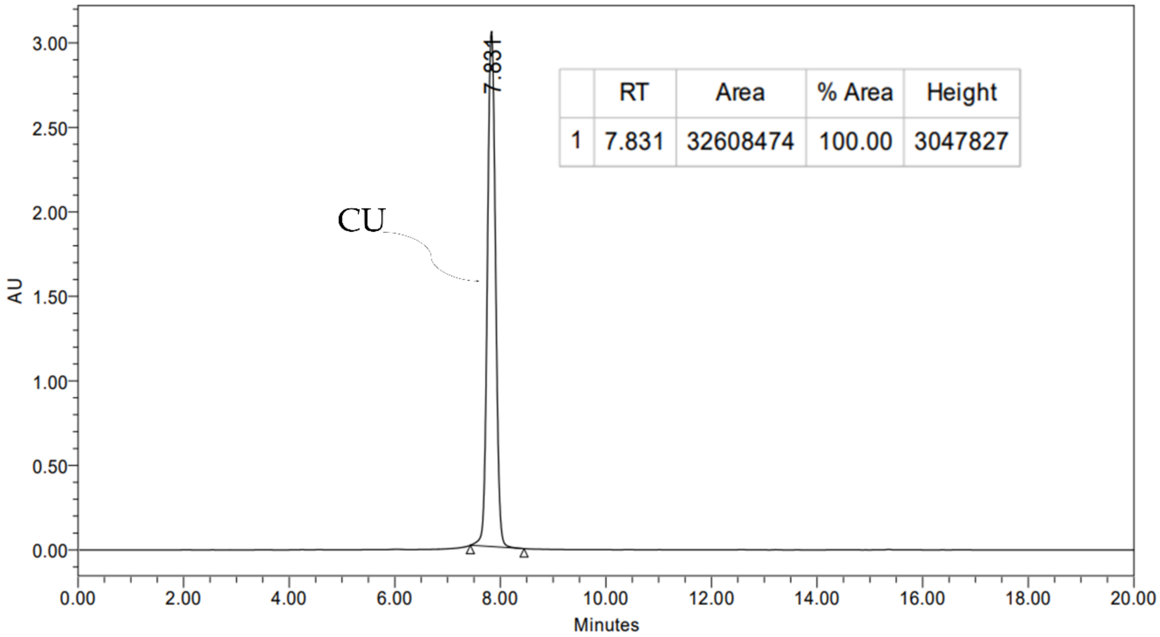

	RT	Area	% Area	Height
1	7.831	32608474	100.00	3047827

Figure S5 HPLC profile of CU at 100 ppm

Table S1 ^{1}H NMR (400 MHz, $CDCl_3$) and ^{13}C NMR (100 MHz, $CDCl_3$) of (1*E*,4Z,6*E*)-1,7-bis(4-ethoxy-3-methoxyphenyl)-5-hydroxyhepta-1,4,6-trien-3-one (**4c)**

No.	δ_H (*J* in Hz)	δ_C
1	7.60, d (15.8)	140.42
2	6.49, d (15.8)	122.48
3	-	183.28
4	5.81, s	101.23
5	-	183.28
6	6.49, d (15.8)	122.48
7	7.60, d (15.8)	140.42
1′ & 1″	-	127.78
2′& 2″	7.08, d (1.7)	110.14
3′& 3″	-	149.37
4′&4″	-	150.55
5′&5″	6.87, d (8.3)	112.22
6′&6″	7.12, dd (8.3, 1.8)	121.90
1‴&1⁗	4.14, q (7.0)	64.58
2OCH_3	3.92, s	56.04
2‴&2⁗	1.49, t (7.0)	14.60

Table S2. Interaction of CU4c in the HDAC active sites.

HDACs	Binding interaction
HDAC1	Gly146 (2.10 Å), Ser148 (2.50 Å, 3.05 Å), Asp181 (2.92 Å, 2.94 Å), Tyr204 (4.09 Å, 5.24Å), Phe205 (2.46 Å), Pro206 (2.55Å, 4.56 Å)
HDAC2	Zn (2.82 Å), Pro106 (4.10 Å, 4.66 Å), His146 (3.98 Å), Gly154 (2.70 Å), Phe155 (4.31 Å), His183 (4.69 Å), Phe210 (4.35 Å, 4.85 Å)
HDAC3	Zn (2.96 Å), Asp93 (2.80 Å), Phe144 (4.24 Å), His172 (3.72 Å, 4.65 Å), Tyr198 (2.18 Å), Phe200 (4.67 Å), Asp225 (2.04 Å, 2.72 Å), Leu266 (4.62 Å), Cys268 (2.97 Å), Tyr298 (4.40 Å)
HDAC8	Gly271 (2.31 Å), Pro273 (4.31 Å, 4.55Å), Ala339 (1.80 Å), Ile348 (2.71 Å, 5.08 Å)
HDAC4	Zn (2.4 Å), Lys20 (2.12 Å), Arg37 (5.32Å), Pro156 (5.33 Å), His159 (2.71 Å), Phe168 (5.36 Å), His198 (4.93 Å), Phe227 (4.42 Å), Leu229 (4.83 Å), Gly330 (1.92 Å, 2.76 Å)
HDAC6	Ser150 (1.84 Å), Asp149 (2.45 Å), His192 (4.99 Å), His193 (4.34 Å), Phe202 (4.06 Å), His232 (3.05 Å, 3.87 Å, 4.38 Å), Trp261 (2.31 Å, 5.41 Å, 5.45 Å), Pro262 (2.31 Å), Tyr363 (2.50 Å),
HDAC7	Zn (2.20 Å), Pro667 (4.73 Å), His669 (4.25 Å), His670 (4.92 Å), Phe679 (4.37 Å), Asp707(2.50 Å), His709 (4.37 Å), Arg731 (3.04 Å, 2.98 Å), Phe737 (4.58 Å)
